# What if you can't sense your enemy… and your enemy is an invasive predator?

**DOI:** 10.1093/conphys/cox011

**Published:** 2017-02-23

**Authors:** Jodie L. Rummer

**Affiliations:** 1 ARC Centre of Excellence for Coral Reef Studies, James Cook University, Townsville, QLD 4811,Australia

It turns out that the lionfish—an invasive fish species that is especially plaguing western Atlantic waters these days—is even more of a threat than we originally thought. Some of its prey species, like small coral reef fishes, cannot identify the lionfish as a threat.

Indeed, the phrase ‘invasive species’ evokes a horrible image to most. An invasive species can take over a non-native habitat, destroy the environment and compete with native plants and animals in its path. For example, feral rabbits, cats and foxes have been the major factor in the disappearance of many of Australia's endemic marsupials. Invasive species can affect humans economically and, in some cases, can impact human health by causing diseases. Not surprisingly, a lot of time, money and brainpower are invested in the prevention, and when protective mechanisms fail, eradication of invasive species in the safest way possible.

Lionfish were accidentally introduced to non-native habitats *via* the aquarium trade and fish hobbyists. Over the past decade, the venomous lionfish has become a prime threat to coral reefs and fisheries. Now, McCormick and Allan (2016) have found something to add to the lionfish's disturbing résumé. They determined that lionfish might also possess some trait that allows them to be even stealthier in their attack mode, thus causing even more damage to coral reef ecosystems. The team found that even when small coral reef damselfish were trained to associate lionfish as a threat through a co-occurrence with damage-released cues—not unlike what fish in the wild would release when injured—the damsels treated lionfish the same as they did a non-threatening butterflyfish. Yet, damsels exhibited increased escape performance upon exposure to a predatory cod. This means that the damsels were misclassifying the lionfish as safe, and therefore were not inclined to instigate the appropriate escape strategy. Something about the lionfish was masking the cue that the damselfish needed to know a threat was in its vicinity.

So, how do we control especially stealthy invasive species? Well, we certainly need to find out more information as to what makes them so ninja-like. Are lionfish emitting something that masks other odours? If so, can we control that mechanism? Otherwise, this trait will only help them to be even more successful at invading pristine habitats and compromising native species.

McCormick and Allan's findings are a good example of the importance of really knowing your enemy. And, with invasive species becoming more and more problematic for coral reef ecosystems, especially on top of all of the other stressors they are facing, it is critical to understand the invasive species before attempting to control it. If we do not, we could end up with another case like the cane toad. Scientists and farmers thought introducing cane toads would help control local pests, but they took over, wiping out important native species with its toxic skin killing most all other animals that try to eat it. At least we can eat lionfish.

**Figure:**
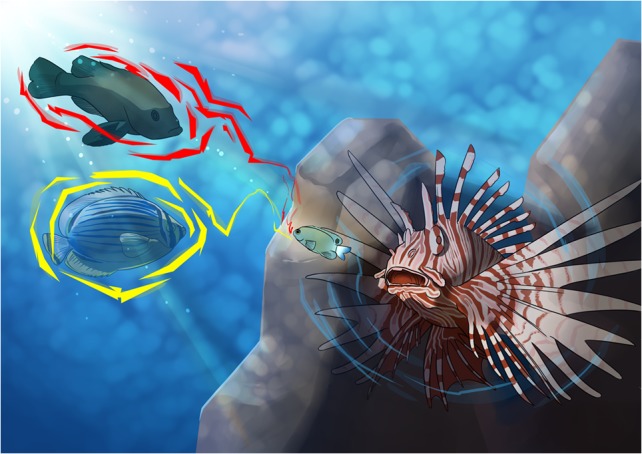

Illustration by Erin Walsh; Email: ewalsh.sci@gmail.com
